# Cathepsin D: A Candidate Link between Amyloid β-protein and Tauopathy in Alzheimer Disease

**Published:** 2021

**Authors:** Caitlin N. Suire, Malcolm A. Leissring

**Affiliations:** 1 Institute for Memory Impairments and Neurological Disorders, University of California, Irvine, Irvine, CA 92697 USA

**Keywords:** Alzheimer disease, Amyloid β-protein, Cathepsin D, Lysosome, Tau

## Abstract

Alzheimer disease (AD) is a debilitating neurodegenerative disorder characterized by extracellular deposition of the amyloid β-protein (Aβ) and intraneuronal accumulation of the microtubule-associated protein, tau. Despite a wealth of experimental and genetic evidence implicating both Aβ and tau in the pathogenesis of AD, the precise molecular links between these two pathological hallmarks have remained surprisingly elusive. Here, we review emerging evidence for a critical nexus among Aβ, tau, and the lysosomal protease cathepsin D (CatD) that we hypothesize may play a pivotal role in the etiology of AD. CatD degrades both Aβ and tau *in vitro*, but the *in vivo* relevance of this lysosomal protease to these principally extracellular and cytosolic proteins, respectively, had remained undefined for many decades. Recently, however, our group found that genetic deletion of CatD in mice results in dramatic accumulation of Aβ in lysosomes, revealing that Aβ is normally trafficked to lysosomes in substantial quantities. Moreover, emerging evidence suggests that tau is also trafficked to the lysosome via chaperone-mediated autophagy and other trafficking pathways. Thus, Aβ, tau and CatD are colocalized in the lysosome, an organelle that shows dysfunction early in AD pathogenesis, where they can potentially interact. Notably, we discovered that Aβ42—the Aβ species most strongly linked to AD pathogenesis—is a highly potent, low-nanomolar, competitive inhibitor of CatD. Taking these observations together, we hypothesize that Aβ42 may trigger tauopathy by competitive inhibition of CatD-mediated degradation of tau—pathogenic forms of tau, in particular. Herein, we review the evidence supporting this hypothesis and explore the implications for the molecular pathogenesis of AD. Future research into these novel mechanistic links among Aβ, tau and CatD promises to expand our understanding of the etiology of AD and could potentially lead to novel therapeutic approaches for combatting this devastating disease of brain and mind.

## Aβ and Tau in Alzheimer Disease

Alzheimer disease (AD) is an increasingly common age-related neurodegenerative disorder, currently affecting ∼44 million people worldwide [1]. Histopathologically, the disease is characterized by progressive accumulation of extracellular deposits of the amyloid β-protein (Aβ), known as plaques, together with intraneuronal aggregates of hyperphosphorylated forms of the microtubule-associated protein, tau, known as neurofibrillary tangles (NFTs) [2]. Despite decades of intensive research within academia and private industry, disease-modifying treatments have not yet emerged. Indeed, several fundamental questions about the molecular pathogenesis of AD remain unresolved.

Among the most fundamental of all unresolved questions within the AD field is precisely how Aβ accumulation triggers NFT formation [3]. A wealth of experimental evidence strongly implicates Aβ mismetabolism in the pathogenesis of AD [4]. For instance, human molecular genetic research has identified hundreds of mutations in 3 separate genes—*amyloid precursor protein* (*App*) and *presenilin-1* and *−2* (*Psen1* and *Psen2*)—that cause familial forms of AD with 100% penetrance [5]. Significantly, all of these mutations affect the production of Aβ in one way or another. *App* mutations, on the one hand, increase the production of all forms of Aβ. *Presenilin* mutations, on the other hand, specifically increase the Aβ42/40 ratio—i.e., the ratio of longer, more aggregation-prone Aβ species, such as Aβ42, and shorter, less amyloidogenic species, such as Aβ40 [5,6]. The presenilins proteins make up the active site of γ-secretase, a proteolytic complex responsible for defining the C-terminus of Aβ during its cleavage from the amyloid precursor protein (APP) [7–9]. The presenilin/γ-secretase complex can cleave APP at any of several sites, resulting in Aβ peptides ranging in length from 37 to 43 amino acids [10]. AD-linked *presenilin* mutations specifically increase the Aβ42/40 ratio by perturbing the function of γ-secretase and, therefore, are believed to be the sole determinant of this critical parameter [11].

NFTs are another invariant feature of AD, emerging somewhat later than Aβ deposits [3]. NFTs are comprised of aggregates of hyperphosphorylated tau that form within the cytosol of neurons [12]. Of note, NFTs are present in several other neurodegenerative diseases [13], in some cases due to genetic mutations in the gene for tau, *Mapt* [14]. These observations suggest that NFT formation is the necessary and most proximal cause of the neuronal cell death common to all these neurodegenerative diseases [13].

The question of how Aβ accumulation leads to NFT formation has constituted an enduring enigma within the AD field. On the one hand, plaques are extracellular, so Aβ has been widely regarded as a secreted protein [15]; indeed, the proteases responsible for the production of Aβ from APP are referred to as “secretases” to reflect this understanding [16]. On the other hand, tau has been regarded as primarily a cytosolic protein [12]. How—and where—then, can extracellular Aβ and intracellular tau interact?

## Lysosomal Aβ and Tau—A Paradigm Shift

In the light of emerging evidence, the long-standing view of Aβ as an exclusively secreted protein, and of tau as an exclusively cytosolic one, has proved to be incorrect. Instead, it has become clear that at least a subset of both proteins co-exist within the lysosome. With respect to Aβ, several observations are relevant. First, within neurons, extracellular Aβ is trafficked via the endolysosomal pathway to lysosomes, where it is degraded by lysosomal proteases [17,18]. Of note, this trafficking is mediated by apolipoprotein E (ApoE), the strongest genetic risk factor for late-onset AD [18,19]. Second, recent findings by our group have demonstrated that a significant portion of newly synthesized Aβ is normally trafficked to the lysosome [20]. Specifically, we found that genetic deletion of cathepsin D (CatD), a lysosomal aspartyl protease discovered more than 40 years ago [21], results in dramatic, ∼3 to ∼4-fold increases in cerebral Aβ by just 3 weeks of age [20]. Notably, the magnitude of these increases exceeds those obtained following genetic deletion of all previously described Aβ-degrading proteases (AβDPs), including the “major” AβDPs neprilysin or insulin-degrading enzyme — or even both simultaneously [20]. Given that CatD is an aspartyl protease, and thus operative only at an acidic pH, it would be predicted that the increases in cerebral Aβ are occurring exclusively within the acidic environment of the lysosome [22]. Confirming this, CatD-null mice exhibit robust deposition of intralysosomal Aβ—endogenous murine Aβ—by just ∼26 days of age [20], something not seen in any other AβDP-null animal [23]. Taken together, these findings strongly imply that a substantial fraction of Aβ is trafficked to lysosomes, where it is normally degraded by CatD and other lysosomal AβDPs such as cathepsin B [24]. Given that the β- and γ-secretases responsible for Aβ production are both aspartyl proteases, too [16], this conclusion is perhaps not too surprising, but there had been little direct evidence prior to the analysis of CatD-null mice.

**Figure 1:**
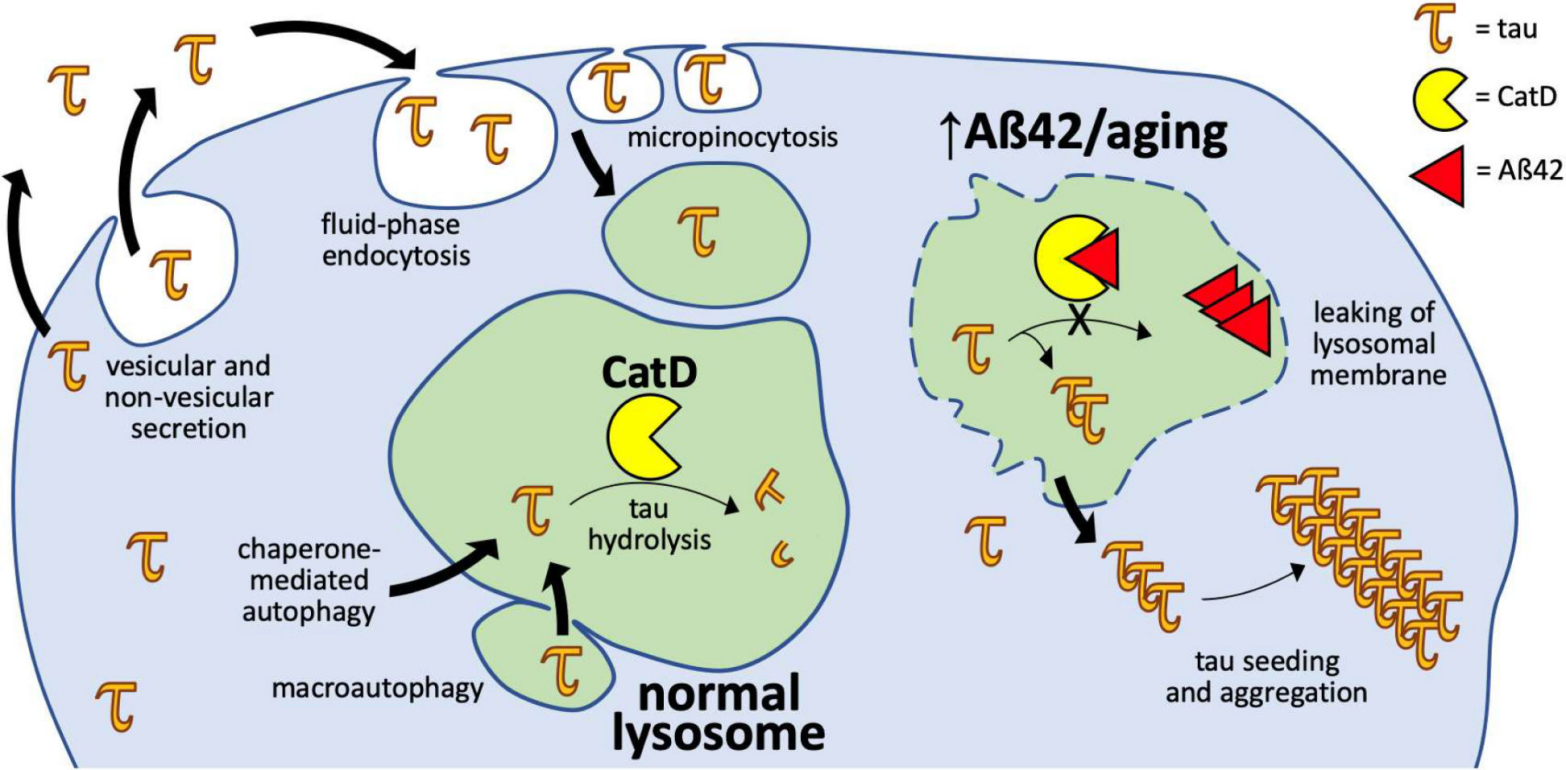
Cellular trafficking pathways linking tau, Aβ42 and CatD. Cartoon illustrating the several pathways by which tau can be trafficked to lysosomes. Within normal lysosomes (*left*) tau is hydrolyzed efficiently by CatD, but in the presence of elevated Aβ42 levels (*right*) CatD is inhibited, preventing tau hydrolysis, and thereby promoting accumulation of tau species. During aging and in AD, the lysosomal membranes become leaky, permitting the efflux of aggregated tau into the cytosol, which can seed the formation of NFTs.

With respect to tau, emerging evidence has also challenged the idea that it is an exclusively cytosolic protein. Tau has long been known to be present in cerebrospinal fluid, and its levels correlate with AD severity, but mechanistically this was initially attributed to non-specific release from dying neurons [25]. Over the past decade, however, a substantial body of research has shown that tau is secreted via multiple vesicular and non-vesicular pathways, not all associated with disease [26] (Figure 1). Because the extracellular space is topologically contiguous with the lumen of the lysosome, these secretion mechanisms render tau capable of eventual interaction with Aβ and CatD. Among several identified trafficking mechanisms, endogenous tau has been shown to be secreted via unconventional protein secretion pathways from primary neurons [26–31]. Once secreted, extracellular tau can enter neurons and other cell types via fluid-phase endocytosis and micropinocytosis [29], whereupon it can be trafficked to the lysosome via conventional mechanisms. Tau can also enter the lysosome directly from the cytosol via both nonselective and selective mechanisms (Figure 1). In the nonselective pathway, lysosomes degrade cytosolic contents in bulk via macroautophagy, wherein cytosolic contents are first enveloped in a membrane that subsequently fuses with the lysosomal membrane to release its contents [32]. The selective pathway, on the other hand, is known as chaperone-mediated autophagy (CMA), wherein substrate proteins directly cross from the cytosol into the lysosome, one at a time [33]. CMA is mediated by a specific targeting motif (KFERQ-like), present within tau, that binds to the cytosolic chaperone, HSC70, which then brings the substrate to the lysosomal surface for internalization [34].

Collectively, these observations overturn conventional thinking about the subcellular localization of Aβ and tau, showing that a significant fraction of both proteins is trafficked to the lysosome.

## Aβ42 Potently Inhibits CatD

Knowing that Aβ and tau can be colocalized in the lysosome, how might they interact? One intriguing possibility is via competition for degradation by CatD. While characterizing Aβ degradation by CatD, our group made the surprising discovery that CatD degrades Aβ42 and Aβ40 with remarkably different kinetics [20]. Most AβDPs hydrolyze Aβ40 and Aβ42 similarly, with Michaelis-Menten constant (K_M_) values in the low- to mid-micromolar range. CatD hydrolyzes Aβ40 with a low-micromolar K_M_, but—in marked contrast—it degrades Aβ42 with a K_M_ value more than two orders of magnitude lower, in the low-nanomolar range, corresponding to a significantly stronger affinity of Aβ42 for CatD [20]. In terms of the turnover number, *k*_cat_, which corresponds to the number of substrate molecules hydrolyzed per unit time by each protease molecule, the difference between Aβ42 and Aβ40 is similarly striking: the *k*_cat_ of Aβ42 degradation by CatD is ∼200-fold slower than that for Aβ40 [20]. In absolute terms, the *k*_cat_ of Aβ42 degradation determined by multiple independent assays was approximately 0.23 min^−1^, *meaning that it takes >4 minutes for each molecule of Aβ42 to be processed by each molecule of CatD* [20].

Taken together, the low K_M_ and *k*_cat_ values of Aβ42 degradation by CatD render Aβ42 a highly potent competitive inhibitor. For example, in competition experiments using a fluorogenic peptide substrate to monitor CatD activity, the IC_50_ of Aβ40 was ∼3 μM, whereas that for Aβ42 was a phenomenally low 0.00097 μM, *meaning that Aβ42 competitively inhibits CatD with subnanomolar potency* [20]. Perhaps more intriguingly, we discovered that the marked differences between Aβ40 and Aβ42 extend to the corresponding α-secretase-derived P3 fragments ending at positions 40 and 42 (e.g., Aβ_17–40_ and Aβ_17–42_ or P3_40_ and P3_42_) [20]. This finding highlights the fact that these differential kinetics occur independently of Aβ aggregation. Moreover, it is notable that P3 fragments are produced in ∼10-fold higher quantities than Aβ [35], substantially increasing the likelihood that these fragments could inhibit CatD at near-physiological levels.

## A New Take on the Aβ42/40 Ratio

As mentioned above, it had long been implicitly assumed that the Aβ42/40 ratio is determined exclusively by the action of the presenilin/γ-secretase complex during the production of Aβ from APP. In CatD-null mice, however, cerebral Aβ42/40 ratios were found to be consistently increased, independent of any effect on APP processing [20]. Our finding that Aβ42 and Aβ40 are processed so differently suggests that, at least in the case of CatD, A42/40 ratios can also be regulated after Aβ production, via differential degradation. Whereas elevated Aβ42/40 ratios are known to be the root cause of familial forms of AD due to *presenilin* mutations [11], this finding suggests that perturbed Aβ catabolism might also affect this key parameter, possibly playing a role in the etiology of late-onset forms of AD.

In terms of the molecular pathogenesis of AD, the ability of Aβ42 and P3_42_ to so potently inhibit CatD suggests another tantalizing, albeit speculative, possibility. Specifically, we hypothesize that the mechanism by which elevated Aβ42/40 (and P3_42/40_) ratios trigger downstream pathological sequelae may, in part, involve competitive inhibition of CatD [20]. In humans and other animals, loss-of-function mutations in CatD lead to multiple forms of neurodegenerative disease [36–38]. Genetic variations in CatD, moreover, have been linked to late-onset AD specifically [39]. In the light of these observations, it seems plausible that dysregulation of CatD by Aβ42 could represent one mechanism linking Aβ accumulation to downstream pathological sequelae.

## Inhibition of Tau Processing by Aβ42

As one of the principal lysosomal proteases, it is no surprise that CatD can degrade tau once it reaches the lysosome [40]. It follows that the accumulation of A42 and P3_42_ could potentially lead to increases in total levels of tau via competitive inhibition of CatD [20]. The situation is actually more complex, however, since CatD can also processes tau proteolytically in several pathologically relevant ways, in some cases producing tau species that are more aggregation-prone and/or directly toxic and in other cases clearing the pathogenic tau species [41–43]. One finding is clear, however: inhibition of CatD and/or dysregulation of lysosomal function increases the levels of multiple neurotoxic tau fragments. In a *drosophila* model expressing human tau, for example, genetic deletion of CatD significantly exacerbated tauopathy [44]. This effect was mediated not by an increase in overall tau levels, but instead by a selective increase in a neurotoxic, caspase-cleaved form of tau [44]. Given that most tau remains cytosolic, the lack of effect on total tau is unsurprising. Nevertheless, this study supports the idea that CatD is involved in the regulation of pathological forms of tau. Regardless of the underlying complexity, the hypothesis that Aβ42 might trigger tauopathy via its ability to potently inhibit CatD seems well substantiated by the existing literature and worthy of further research.

## Conclusion

In conclusion, we hypothesize that Aβ42—the Aβ species most strongly linked to AD pathogenesis—can trigger tauopathy by competitively inhibiting the clearance of neurotoxic tau species by CatD within the lysosome. As reviewed above, accruing evidence supports the idea that significant quantities of both tau and Aβ are normally trafficked to the lysosome and degraded by CatD. The recent finding that Aβ42 and P3_42_ potently inhibit CatD [20] suggests a discrete molecular mechanism linking Aβ42 accumulation to the accumulation of neurotoxic tau species. Since lysosomes become dysfunctional and leaky in aging generally [45], and in response to Aβ42 specifically [46], accumulated neurotoxic tau fragments could reach the cytosol, where they might seed the formation of NFTs and, via this mechanism and others, contribute to cell death (Figure 1). In addition to being well supported by a range of evidence, reviewed herein, this hypothesis has the advantage of being specific and readily testable. Given the enormous economic and societal burden exacted by AD, future research into this hypothetical pathogenic mechanism seems highly warranted.

## References

[1] Alzheimer’s Association. 2020 Alzheimer’s disease facts and figures. Alzheimer’s & Dementia : The Journal of the Alzheimer’s Association. 2020 Mar 10. 10.1002/alz.12068 32157811

[2] Sengoku R Aging and Alzheimer’s disease pathology. Neuropathology. 2020 Feb;40(1):22–9. 31863504 10.1111/neup.12626

[3] Gallardo G, Holtzman DM. Amyloid-β and tau at the crossroads of Alzheimer’s disease. Tau Biology. 2019:187203. 10.1007/978-981-32-9358-8_16 32096039

[4] Selkoe DJ, Hardy J. The amyloid hypothesis of Alzheimer’s disease at 25 years. EMBO Molecular Medicine. 2016 Jun;8(6):595–608. 27025652 10.15252/emmm.201606210 PMC4888851

[5] Cacace R, Sleegers K, Van Broeckhoven C. Molecular genetics of early-onset Alzheimer’s disease revisited. Alzheimer’s & Dementia. 2016 Jun 1;12(6):733–48. 10.1016/j.jalz.2016.01.012 27016693

[6] Scheuner D, Eckman C, Jensen M, Song X, Citron M, Suzuki N, Secreted amyloid β–protein similar to that in the senile plaques of Alzheimer’s disease is increased in vivo by the presenilin 1 and 2 and APP mutations linked to familial Alzheimer’s disease. Nature Medicine. 1996 Aug;2(8):864–70. 10.1038/nm0896-864 8705854

[7] Kimberly WT, LaVoie MJ, Ostaszewski BL, Ye W, Wolfe MS, Selkoe DJ. γ-Secretase is a membrane protein complex comprised of presenilin, nicastrin, aph-1, and pen-2. Proceedings of the National Academy of Sciences. 2003 May 27;100(11):6382–7. 10.1073/pnas.1037392100 PMC164455 12740439

[8] De Strooper B, Iwatsubo T, Wolfe MS. Presenilins and γ-secretase: structure, function, and role in Alzheimer disease. Cold Spring Harbor Perspectives in Medicine. 2012 Jan 1;2(1):a006304. 22315713 10.1101/cshperspect.a006304 PMC3253024

[9] Bai XC, Yan C, Yang G, Lu P, Ma D, Sun L, An atomic structure of human γ-secretase. Nature. 2015 Sep;525(7568):212–7. 26280335 10.1038/nature14892 PMC4568306

[10] Wolfe MS. The γ-secretase complex: membrane-embedded proteolytic ensemble. Biochemistry. 2006 Jul 4;45(26):7931–9. 16800619 10.1021/bi060799c

[11] Haass C, De Strooper B. The presenilins in Alzheimer’s disease--proteolysis holds the key. Science. 1999 Oct 29;286(5441):916–9. 10542139 10.1126/science.286.5441.916

[12] Kosik KS. Alzheimer’s disease: a cell biological perspective. Science. 1992 May 8;256(5058):780–3. 1589757 10.1126/science.1589757

[13] Goedert M Tau filaments in neurodegenerative diseases. FEBS Letters. 2018 Jul 1;592(14):2383–91. 29790176 10.1002/1873-3468.13108

[14] Wolfe MS. Tau mutations in neurodegenerative diseases. Journal of Biological Chemistry. 2009 Mar 6;284(10):6021–5. 18948254 10.1074/jbc.R800013200

[15] Haass C, Hung AY, Schlossmacher MG, Oltersdorf T, Teplow DB, Selkoe DJ. Normal cellular processing of the β-amyloid precursor protein results in the secretion of the amyloid β peptide and related molecules. Annals of the New York Academy of Sciences. 1993 Sep;695(1):109–16. 8239267 10.1111/j.1749-6632.1993.tb23037.x

[16] De Strooper B, Vassar R, Golde T. The secretases: enzymes with therapeutic potential in Alzheimer disease. Nature Reviews Neurology. 2010 Feb;6(2):99–107. 20139999 10.1038/nrneurol.2009.218 PMC2879045

[17] Jiang Q, Lee CD, Mandrekar S, Wilkinson B, Cramer P, Zelcer N, ApoE promotes the proteolytic degradation of Aβ. Neuron. 2008 Jun 12;58(5):681–93. 18549781 10.1016/j.neuron.2008.04.010 PMC2493297

[18] Li J, Kanekiyo T, Shinohara M, Zhang Y, LaDu MJ, Xu H, Differential regulation of amyloid-β endocytic trafficking and lysosomal degradation by apolipoprotein E isoforms. Journal of Biological Chemistry. 2012 Dec 28;287(53):44593–601. 23132858 10.1074/jbc.M112.420224 PMC3531774

[19] Holtzman DM, Herz J, Bu G. Apolipoprotein E and apolipoprotein E receptors: normal biology and roles in Alzheimer disease. Cold Spring Harbor Perspectives in Medicine. 2012 Mar 1;2(3):a006312. 22393530 10.1101/cshperspect.a006312 PMC3282491

[20] Suire CN, Abdul-Hay SO, Sahara T, Kang D, Brizuela MK, Saftig P, Cathepsin D regulates cerebral Aβ42/40 ratios via differential degradation of Aβ42 and Aβ40. Alzheimer’s Research & Therapy. 2020 Dec;12(1):1–3. 10.1186/s13195-020-00649-8 PMC7339583 32631408

[21] Westley B, Rochefort H. A secreted glycoprotein induced by estrogen in human breast cancer cell lines. Cell. 1980 Jun 1;20(2):353–62. 7388945 10.1016/0092-8674(80)90621-2

[22] Benes P, Vetvicka V, Fusek M. Cathepsin D—many functions of one aspartic protease. Critical Reviews in Oncology/Hematology. 2008 Oct 1;68(1):12–28. 18396408 10.1016/j.critrevonc.2008.02.008 PMC2635020

[23] Leissring MA. Proteolytic degradation of the amyloid β-protein: the forgotten side of Alzheimer’s disease. Current Alzheimer Research. 2006 Dec 1;3(5):431–5. 17168642 10.2174/156720506779025206

[24] Sun B, Zhou Y, Halabisky B, Lo I, Cho SH, MuellerSteiner S, Cystatin C-cathepsin B axis regulates amyloid beta levels and associated neuronal deficits in an animal model of Alzheimer’s disease. Neuron. 2008 Oct 23;60(2):247–57. 18957217 10.1016/j.neuron.2008.10.001 PMC2755563

[25] Johnson GV, Seubert P, Cox TM, Motter R, Brown JP, Galasko D. The τ protein in human cerebrospinal fluid in Alzheimer’s disease consists of proteolytically derived fragments. Journal of neurochemistry. 1997 Jan;68(1):430–3. 8978756 10.1046/j.1471-4159.1997.68010430.x

[26] Pernègre C, Duquette A, Leclerc N. Tau secretion: good and bad for neurons. Frontiers in Neuroscience. 2019 Jun 26;13:649. 31293374 10.3389/fnins.2019.00649 PMC6606725

[27] Brunello CA, Merezhko M, Uronen RL, Huttunen HJ. Mechanisms of secretion and spreading of pathological tau protein. Cellular and Molecular Life Sciences. 2020 May;77(9):1721–44. 31667556 10.1007/s00018-019-03349-1 PMC7190606

[28] Merezhko M, Brunello CA, Yan X, Vihinen H, Jokitalo E, Uronen RL, Secretion of tau via an unconventional non-vesicular mechanism. Cell Reports. 2018 Nov 20;25(8):2027–35. 30463001 10.1016/j.celrep.2018.10.078

[29] Evans LD, Wassmer T, Fraser G, Smith J, Perkinton M, Billinton A, Extracellular monomeric and aggregated tau efficiently enter human neurons through overlapping but distinct pathways. Cell Reports. 2018 Mar 27;22(13):3612–24. 29590627 10.1016/j.celrep.2018.03.021 PMC5896171

[30] Mohamed NV, Herrou T, Plouffe V, Piperno N, Leclerc N. Spreading of tau pathology in Alzheimer’s disease by cell-to-cell transmission. European Journal of Neuroscience. 2013 Jun;37(12):1939–48. 23773063 10.1111/ejn.12229

[31] Cirone M Perturbation of bulk and selective macroautophagy, abnormal UPR activation and their interplay pave the way to immune dysfunction, cancerogenesis and neurodegeneration in ageing. Ageing Research Reviews. 2020 Mar 1;58:101026. 32018054 10.1016/j.arr.2020.101026

[32] Kaushik S, Cuervo AM. The coming of age of chaperone-mediated autophagy. Nature Reviews Molecular Cell Biology. 2018 Jun;19(6):365–81. 29626215 10.1038/s41580-018-0001-6 PMC6399518

[33] Fontaine SN, Zheng D, Sabbagh JJ, Martin MD, Chaput D, Darling A, Trotter JH, Stothert AR, Nordhues BA, Lussier A, Baker J. DnaJ/Hsc70 chaperone complexes control the extracellular release of neurodegenerative-associated proteins. The EMBO Journal. 2016 Jul 15;35(14):1537–49. 27261198 10.15252/embj.201593489 PMC4946142

[34] Haass C, Hung AY, Schlossmacher MG, Teplow DB, Selkoe DJ. beta-Amyloid peptide and a 3-kDa fragment are derived by distinct cellular mechanisms. Journal of Biological Chemistry. 1993 Feb 15;268(5):3021–4. 8428976

[35] Shacka JJ, Klocke BJ, Young C, Shibata M, Olney JW, Uchiyama Y, Saftig P, Roth KA. Cathepsin D deficiency induces persistent neurodegeneration in the absence of Bax-dependent apoptosis. Journal of Neuroscience. 2007 Feb 21;27(8):2081–90. 17314303 10.1523/JNEUROSCI.5577-06.2007 PMC6673541

[37] Steinfeld R, Reinhardt K, Schreiber K, Hillebrand M, Kraetzner R, Brück W, Cathepsin D deficiency is associated with a human neurodegenerative disorder. The American Journal of Human Genetics. 2006 Jun 1;78(6):988–98. 16685649 10.1086/504159 PMC1474096

[38] Tyynelä J, Sohar I, Sleat DE, Gin RM, Donnelly RJ, Baumann M, A mutation in the ovine cathepsin D gene causes a congenital lysosomal storage disease with profound neurodegeneration. The EMBO Journal. 2000 Jun 15;19(12):2786–92. 10856224 10.1093/emboj/19.12.2786 PMC203370

[39] Davidson Y, Gibbons L, Pritchard A, Hardicre J, Wren J, Tian J, Genetic associations between cathepsin D exon 2 C→ T polymorphism and Alzheimer’s disease, and pathological correlations with genotype. Journal of Neurology, Neurosurgery & Psychiatry. 2006 Apr 1;77(4):515–7. 16543533 10.1136/jnnp.2005.063917 PMC2077521

[40] Kenessey A, Nacharaju P, Ko LW, Yen SH. Degradation of tau by lysosomal enzyme cathepsin D: implication for Alzheimer neurofibrillary degeneration. Journal of Neurochemistry. 1997 Nov;69(5):2026–38. 9349548 10.1046/j.1471-4159.1997.69052026.x

[41] Wang Y, Martinez-Vicente M, Krüger U, Kaushik S, Wong E, Mandelkow EM, Tau fragmentation, aggregation and clearance: the dual role of lysosomal processing. Human Molecular Genetics. 2009 Nov 1;18(21):4153–70. 19654187 10.1093/hmg/ddp367 PMC2758146

[42] Bi X, Haque TS, Zhou J, Skillman AG, Lin B, Lee CE, Novel cathepsin D inhibitors block the formation of hyperphosphorylated tau fragments in hippocampus. Journal of Neurochemistry. 2000 Apr;74(4):1469–77. 10737603 10.1046/j.1471-4159.2000.0741469.x

[43] Bednarski E, Lynch G. Cytosolic proteolysis of τ by cathepsin D in hippocampus following suppression of cathepsins B and L. Journal of Neurochemistry. 1996 Nov;67(5):1846–55. 8863489 10.1046/j.1471-4159.1996.67051846.x

[44] Khurana V, Elson-Schwab I, Fulga TA, Sharp KA, Loewen CA, Mulkearns E, Lysosomal dysfunction promotes cleavage and neurotoxicity of tau in vivo. PLoS Genet. 2010 Jul 15;6(7):e1001026. 20664788 10.1371/journal.pgen.1001026 PMC2904797

[45] Cataldo AM, Hamilton DJ, Barnett JL, Paskevich PA, Nixon RA. Properties of the endosomal-lysosomal system in the human central nervous system: disturbances mark most neurons in populations at risk to degenerate in Alzheimer’s disease. Journal of Neuroscience. 1996 Jan 1;16(1):186–99. 8613784 10.1523/JNEUROSCI.16-01-00186.1996 PMC6578706

[46] Yang AJ, Chandswangbhuvana D, Margol L, Glabe CG. Loss of endosomal/lysosomal membrane impermeability is an early event in amyloid Aβ1–42 pathogenesis. Journal of Neuroscience Research. 1998 Jun 15;52(6):691–8. 9669318 10.1002/(SICI)1097-4547(19980615)52:6<691::AID-JNR8>3.0.CO;2-3

